# Investigation of possible association between Ser9Gly polymorphism of the D3 dopaminergic receptor gene and response to typical antipsychotics in patients with schizophrenia

**DOI:** 10.1590/S1516-31802006000300013

**Published:** 2006-05-04

**Authors:** Quirino Cordeiro, Karen Miguita, Elisabete Miracca, Hélio Elkis, Homero Vallada

**Keywords:** Pharmacogenetics, Dopamine, Association, Genetic polymorphism, Schizophrenia, Farmacogenética, Dopamina, Associação, Polimorfismo genético, Esquizofrenia

## Abstract

Typical antipsychotics have a high affinity for dopamine receptors. It is therefore of interest to investigate such loci in pharmacogenetic studies on psychosis. We investigated the hypothesis that Ser9Gly polymorphism of the DRD3 gene may play a role in the differences in individual response to typical antipsychotics between schizophrenic patients. The sample was composed of 53 good responders and 59 poor ones. No significant differences between the good and poor responders were found in the allelic distribution (good responders: Ser9 61.32%, Gly9 38.67%; poor responders: Ser9 64.40%, Gly9 35.59%; odds ratio, OR = 0.88, 0.49 < OR < 1.56; χ^2^ = 0.23, 1 degree of freedom, df, p = 0.63) and genotype distribution (good responders: Ser9/Ser9 37.73%, Ser9/Gly9 47.16%, Gly9/Gly9 15.09%; poor responders: Ser9/Ser9 42.37%, Ser9/Gly9 44.06%, Gly9/Gly9 13.55%; χ^2^ = 0.25, 2 df, p = 0.88). Nor was there any association with homozygosity (good responders: homozygous: 52.82%, heterozygous: 47.16%; poor responders: homozygous: 55.92%, heterozygous: 44.06%; odds ratio, OR = 0.88, 0.39 < OR < 1.99; χ^2^ = 0.11, 1 df, p = 0.74). The results did not support the hypothesis that Ser9Gly polymorphism of the DRD3 gene influences the response to typical antipsychotics in our sample of schizophrenics.

## RESUMO

Antipsicóticos típicos apresentam alta afinidade pelos receptores dopaminérgicos, que são, portanto, regiões de interesse para os estudos de farmacogenética das psicoses. No presente estudo, investigamos a hipótese de que o polimorfismo Ser9Gly do gene do DRD3 possa exercer um papel na diferença de resposta inter-individual ao uso de antipsicóticos típicos em pacientes com esquizofrenia. Nossa amostra foi composta por 53 pacientes bons respondedores e 59 maus respondedores. Não houve diferenças nas distribuições alélicas (bons respondedores: Ser9 61,32%, Gly9 38,67%; maus respondedores: Ser9 64,40%, Gly9 35,59%; *odds ratio*, OR = 0,88, 0,49 < OR < 1,56; χ^2^ = 0,23, 1 *degree of freedom*, d.f., p = 0,63) e genotípica (bons respondedores: Ser9/Ser9 37,73%, Ser9/Gly9 47,16%, Gly9/Gly9 15,09%; maus repondedores: Ser9/Ser9 42,37%, Ser9/Gly9 44,06%, Gly9/Gly9 13,55%; χ^2^ = 0,25, 2 d.f., p = 0,88) entre os grupos. Não houve também associação com homozigosidade (bons respondedores: homozigotos: 52,82%, heterozigotos: 47,16%; maus repondedores: homozigotos: 55,92%, heterozigotos: 44,06%; odds ratio, OR = 0,88, 0,39 < OR < 1,99); χ^2^ = 0,11, 1 d.f., p = 0,74). Os resultados não dão suporte à hipótese de que o polimorfismo Ser9Gly do gene do DRD3 possa influenciar a resposta terapêutica aos antipsicóticos típicos na nossa amostra de pacientes com esquizofrenia.

Dear Editor,

Failure to respond to medication is a common problem in psychiatry. Around 30% of the patients with schizophrenia will fail to improve on antipsychotic treatment.^1^ There are three broad reasons for treatment failure: lack of compliance, adverse drug reaction, and simple lack of efficacy. Individual genetic differences clearly play a role in determining both clinical responses to medications, as well as the adverse side effects experienced. However, few genetic factors have been identified and the reasons why some patients fail to respond clinically, despite taking therapeutic doses of medication, remain largely unknown.^2^ Treatment failure may be pharmacokinetic in origin, where the drug concentration is outside the therapeutic window, or pharmacodynamic, where the effect of the drugs on the receptor and downstream signaling system pathway is impaired.

Many pharmacogenetic studies have been performed on atypical antipsychotics, in particular clozapine, but there is less data on responses to typical antipsychotics.^3^ However, the study of typical antipsychotics should not be neglected as they have similar efficacy to atypical drugs and continue to be important in developing countries, for economic reasons.

In the present study, we investigated the hypothesis that Ser9Gly polymorphism of the D3 dopaminergic receptor (DRD3) gene may play a role in the inter-individual difference in response to typical antipsychotic drugs, using schizophrenic patients who have undergone long-term treatment with these drugs.

We analyzed 112 subjects, who met the Diagnostic and Statistical Manual of Mental Disorders, Fourth Edition (DSM-IV) criteria for schizophrenia and were under treatment at the Institute of Psychiatry, Hospital das Clínicas, Faculdade de Medicina da Universidade de São Paulo. Clinical response was retrospectively assessed after medical notes had been evaluated.

Response to antipsychotics was defined according to Kane's criteria for treatment resistance,^4^ i.e. patients who had failed to respond clinically to at least three different typical antipsychotics in two different classes over the last five years were considered to be poor responders. There were 53 good responders and 59 poor responders. Clinical response criteria were based on the presence or absence of hallucinations and/or delusions and the commonest typical antipsychotics used were chlorpromazine, thioridazine and haloperidol. There were no significant differences relating to ethnic, demographic and clinical characteristics between the groups. Ethnic background was assessed based on phenotypic characteristics (Caucasian, African, African-Brazilian, or Asian). The investigators who evaluated the patients’ clinical responses were blind to the genotyping results. Patients who discontinued the antipsychotics because of side effects were excluded from the study.

Genomic DNA was extracted from venous blood and used as a template for polymerase chain reaction (PCR) amplification. Ser9Gly polymorphism was examined using primers and PCR conditions described previously by Lannfelt et al.^5^ Statistical differences between the groups of good and poor responders were tested by using the standard chi-squared test. The level of statistical significance was set at p < 0.05.

No significant differences between the good and poor responders were found in the allelic distribution (good responders: Ser9 61.32%, Gly9 38.67%; poor responders: Ser9 64.40%, Gly9 35.59%; odds ratio, OR = 0.88, 0.49 < OR < 1.56; χ^2^ = 0.23, 1 degree of freedom, df, p = 0.63) and genotype distribution (good responders: Ser9/Ser9 37.73%, Ser9/Gly9 47.16%, Gly9/Gly9 15.09%; poor responders: Ser9/Ser9 42.37%, Ser9/Gly9 44.06%, Gly9/Gly9 13.55%; χ^2^ = 0.25, 2 df, p = 0.88) (Table 1). Nor was there any association with homozygosity (good responders: homozygous: 52.82%, heterozygous: 47.16%; poor responders: homozygous: 55.92%, heterozygous: 44.06%; odds ratio, OR = 0.88, 0.39 < OR < 1.99; χ^2^ = 0.11, 1 df, p = 0.74).

**Table 1.** Distribution of Ser9Gly gene polymorphism genotypes in good and poor responders to typical antipsychotics

	Good responders	Poor responders	Χ^2^	p-value
Alleles				
Ser9	65 (61.32)	76 (64.41)	0.23	0.63
Gly9	41 (38.68)	42 (35.59)		
**Total**	**106 (100)**	**118 (100)**		
Genotypes				
Ser9/Ser9	20 (37.74)	25 (42.37)		
Ser9/Gly9	25 (47.17)	26 (44.07)	0.25	0.88
Gly9/Gly9	8 (15.09)	8 (13.56)		
**Total**	**53 (100)**	**59 (100)**		

Thus, using a Brazilian sample of long-term treated schizophrenic outpatients, we have described a lack of association between the response to typical antipsychotics and Ser9Gly polymorphism of the DRD3 gene.

Although this polymorphism has been intensively studied, the results still remain inconclusive today. In a review paper, Scharfetter^6^ indicated a trend of association between the Gly9 allele and better response to anti-psychotic treatment in general. However, a meta-analysis performed by Jönsson et al.^7^ found an interesting pattern of association, showing that in studies involving patients who were treated with atypical antipsychotics, the Gly9 allele was more frequently reported to be associated with good response and, in studies that included patients receiving typical antipsychotics, the Ser9 allele seemed to be correlated with better treatment response. These results were confirmed recently by Szekeres et al.,^8^ who found that the Ser9 allele and Ser9/Ser9 genotype were more frequent in non-responding schizophrenic patients using atypical antipsychotics (clozapine, olanzapine, quetiapine and risperidone). However these results may be due to the fact that the patients treated, especially those using clozapine (an atypical antipsychotic), were mainly recruited from among former non-responders to typical antipsychotics. Therefore, in this selected subgroup, the Gly9 allele favored better response. This situation may be a source of bias in the hypothesized pattern of association between the different types of antipsychotics and Ser9Gly polymorphism of the DRD3 gene.^9^ The fact is that the Gly9 allele of the DRD3 gene may be associated with the response to clozapine but, in studies in which the choice of antipsychotics is not restricted, the role of this polymorphism is unclear.^10^

We are aware of methodological weaknesses in our investigation: specifically, the retrospective definition of drug response, the use of more than one typical antipsychotic in the patients and the possibility of population stratification. However, even though retrospective, we believe that the analysis of response based on Kane's criteria^4^ is robust because of the long period of treatment on typical drugs (at least five years). The fact that patients were given a variety of typical drugs is unlikely to be a confounding factor, since their primary mechanism of action is similar. The blood levels of the antipsychotic drugs used by the patients were not assessed, but the fact that the patients had undergone long-term treatment with these drugs in our outpatient units diminishes the chance of noncompliance detection.

In conclusion, the results from our work do not support the hypothesis that Ser9Gly polymorphism of the DRD3 gene influences the response to typical antipsychotics, in our Brazilian sample of patients with schizophrenia. However, further studies will be needed in order to elucidate this issue, especially studies involving large samples, such as meta-analysis investigations, with the aim of providing psychiatrists with a biological rationale for targeted antipsychotic treatment.^11^
